# Study of the Use of Antinematode Drugs, Mebendazole and Pyrantel, in Galicia (Spain) from 2016 to 2020

**DOI:** 10.1155/2022/7792006

**Published:** 2022-09-13

**Authors:** S. Vázquez- Prieto, A. Vaamonde, E. Paniagua

**Affiliations:** ^1^Universidad de Los Lagos, Osorno, Chile; ^2^Vicerrectoría de Investigación y Postgrado, Universidad Católica del Maule, Talca, Chile; ^3^Departamento de Estadística e Investigación Operativa, Facultad de Ciencias Económicas y Empresariales, Universidad de Vigo, 36310 Vigo, Spain; ^4^Laboratorio de Parasitología, Departamento de Microbiología y Parasitología, Facultad de Farmacia, Universidad de Santiago de Compostela, Campus Vida, 15782 Santiago de Compostela, Spain; ^5^Instituto de Investigación en Análisis Químicos y Biológicos (IAQBUS), Universidad de Santiago de Compostela, Santiago de Compostela, Spain

## Abstract

To the best of our knowledge, there is no study on the use of drugs focused on the consumption of antinematode drugs in any region of the world. In the present study, we analyzed and evaluated the use of mebendazole and pyrantel in the provinces of Galicia (Spain), as well as described the variability of the consumption of both drugs between these provinces from 2016 to 2020. A descriptive, cross-sectional, and retrospective study of the consumption of these drugs, expressed in defined daily dose per 1000 inhabitants per day (DHD), was carried out. The DHD values for both drugs were small, although clearly higher, both on average and in variability, in the case of mebendazole. The difference in the mean DHD between both drugs and the geographical differences observed was statistically significant. The seasonal differences were statistically significant for both active principles, with lower values in summer. The active principle most consumed in all the provinces and years was mebendazole. The main consequence of the excessive use of this drug compared to pyrantel may be the increased risk of the development of resistance and of therapeutic failure, as well as the consequent limitation of pharmacological options in the future.

## 1. Introduction

In drug utilization studies (DUS), the quantitative and qualitative aspects of the determinants of drug use and their effects are analyzed, either in specific patients or in the general population [1]. Therefore, based on the results obtained, information can be acquired on the actual use of medications and their practical consequences, as well as achieving optimal therapeutic practice, since once a problem in the use of medications has been detected, interventions to solve it can be developed [1, 2]. However, to systematize and compare the data obtained in this type of study, a uniform classification of drugs is necessary, applicable in all countries, and stable over time, as well as the use of appropriate quantitative measurement parameters. Thus, the World Health Organization (WHO) recommends applying the anatomical, therapeutic, and chemical classification (ATC) and the use of the defined daily dose (DDD) as the unit of measurement for drug consumption [2].

To our knowledge, no DUS has been carried out focused on the consumption of antinematodes in any region of the world. This lack is particularly striking if one take into account that knowledge of this type is essential to be able to apply improvement strategies and reflect on the determinants and levels of prescription, with the aim of containing the development of possible resistance [3]. In the present study, we analyzed and evaluated the use of antinematode drugs (mebendazole and pyrantel) in the four provinces of Galicia (Spain), as well as to described the variability of the consumption of this type of drugs between these provinces in the period between 2016 and 2020. The diseases treated with these drugs are not notifiable, so knowledge of the epidemiological situation of these infections in Galicia is difficult to establish since there are no conclusive data on their incidence. However, references in the Spanish literature confirm that these diseases are an important problem both in terms of their prevalence and medical consequences in the country [4–9].

## 2. Materials and Methods

A descriptive, cross-sectional and retrospective study of the consumption of antinematode drugs (group P02C of the ATC classification) of the four provinces of Galicia in the period between 2016 and 2020 was carried out. The study of the consumption of albendazole was excluded since its authorized therapeutic indication in Spain is exclusively the treatment of hydatidosis and neurocysticercosis, both diseases caused by cestodes.

The consumption information was obtained through the General Sub-Directorate of Pharmacy of the Galician Health Service (SERGAS) from the monthly billing database of official medical prescriptions. The data referring to the number of containers of antinematode drugs dispensed in the Galician pharmacy offices were collected by SERGAS between January 1, 2016, and December 31, 2020.

The measure of consumption was the defined daily dose (DDD). As an indicator of consumption, the number of DDD per 1000 inhabitants per day (DHD) was calculated, according to the following formula [10]:

(1) $$\begin{array}{c} \text{DHD}=\frac{\text{no}.\text{of DDD}\times 1000}{\text{population}\times t}, \end{array}$$

where *t* corresponds to the days of each month of the year. The population data were taken from the official figures registered in the database of the Galician Institute of Statistics [11]. The DHD parameter provides a rough idea of the volume of the population treated daily with a usual dose of a given drug [1]. For the information processing, a database was created in the Excel program. The statistical analysis was carried out with the free-use statistical program R version 4.0.3. The means and 95% confidence intervals (95% CI) were analyzed. The difference between the means of DHD between both drugs was calculated by applying Welch's *t*-test. The comparison of consumption between provinces was carried out using the non-parametric Kruskal-Wallis test. In general, the 0.05 level of significance was used.

## 3. Results

**Figure 1:**
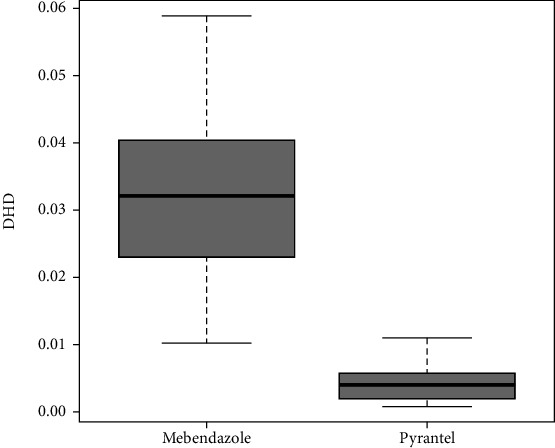
Distribution by active principle.

DHD values for both drugs were small, ranging from 0.001 to 0.059. DHD values were clearly higher, both on average and in variability, in the case of mebendazole (Figure 1). The mean of mebendazole and pyrantel was 0.032 and 0.004, respectively, with the 95% CI for the difference of means 0.026–0.029. The difference in the mean DHD between both drugs was statistically significant (*t* = 38.998; *P* < 0.001). The mean values and standard deviations by active principle and province are described in Table 1.

**Figure 2:**
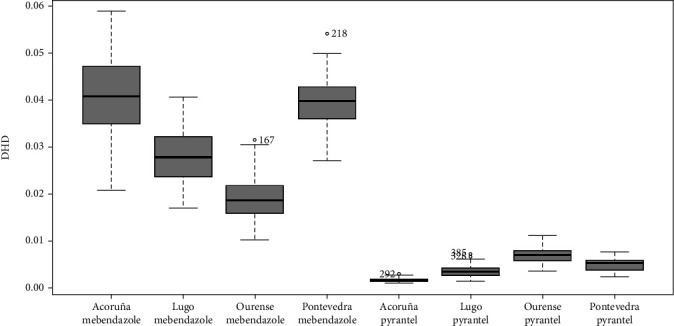
Distribution by province and active principle.

The distribution by province and active principle can be seen in Figure 2. The greatest discrepancy between the two occurred in A Coruña and the least in Ourense. The geographical differences observed were statistically significant: mebendazole (*P* < 0.001) and pyrantel (*P* < 0.001).

**Figure 3:**
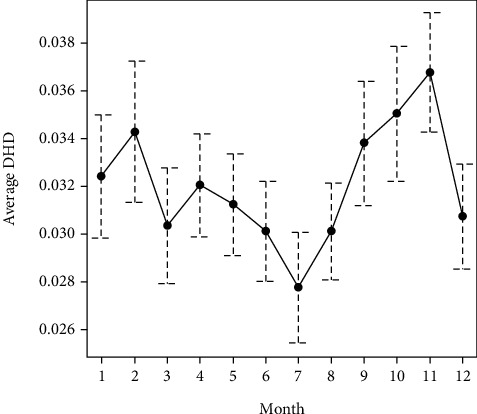
Mean and seasonal variability of DHD mebendazole.

**Figure 4:**
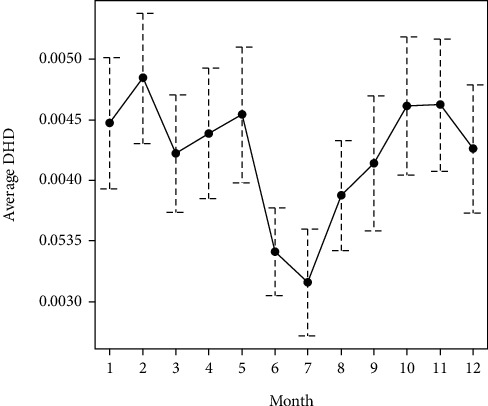
Mean and seasonal variability of DHD pyrantel.

In relation to the temporal evolution, the graphs of the means indicated certain seasonality, with lower values in summer (Figures 3 and 4). In these graphs, you can see the mean and a confidence interval for each month; when the intervals do not overlap (July and November, for example), the difference between those months was statistically significant.

Considering the four seasons (Table 2), the Kruskal-Wallis test indicated statistically significant seasonal differences for both active principles: mebendazole (*P* = 0.042) and pyrantel (*P* = 0.038).

## 4. Discussion

To the best of our knowledge, this is the first DUS focused on the consumption of the mebendazole and pyrantel conducted anywhere in the world. To carry out this study, the international technical unit of measurement recommended by the WHO was used (DDD). Its use allows comparative studies of consumption in different geographical areas and within the same area in different periods of time, regardless of differences or changes in prices or presentations. However, DDD also has certain limitations. Thus, on many occasions, it does not correspond to the prescribed daily dose and/or the amount of medicine consumed by the patient in practice. On the other hand, it does not reflect the indications for which the drugs are used and, sometimes, the same drug may have different doses for different indications [1, 2, 12].

The realization and publication of DUS have enormous importance since they allow us to know the use of drugs in real practice and identify problems related to their use in order to later design intervention strategies that lead to a more effective, safe use and efficient medication [2, 13]. In this study, the results show that mebendazole was much more used than pyrantel in the four provinces of Galicia in the period between 2016 and 2020. A possible explanation for this finding could be that academic training, together with the sources of information available to doctors in their professional practice, generated knowledge and attitudes towards this particular drug, conditioning the prescription practices [14]. From a public health perspective, the high consumption of mebendazole compared to pyrantel in Galicia may contribute to a higher probability of developing drug resistance [15–18]. The risk of serious side effects, including neutropenia and/or thrombocytopenia, and the possible relationship between the simultaneous use of mebendazole and metronidazole and the appearance of Stevens-Johnson syndrome/toxic epidermal necrolysis are also factors to consider for a careful use of mebendazole [19, 20].

The results obtained suggest the existence of a specific geographical pattern, since it is observed that the Atlantic provinces (A Coruña and Pontevedra) present a figure for global consumption of antinematode drugs higher than the inland provinces (Lugo and Ourense). In this sense, it should be noted that the incidence of diseases caused by nematodes is related to the climate and that the areas to the west of the community of Galicia have, in general, milder temperatures. In accordance with this, a certain seasonality was also observed, with higher consumption of antinematode drugs in fall and winter, seasons in which the high humidity and mild temperatures, typical of the region as a whole, favor the development of parasites. The higher volume of mebendazole consumption observed in A Coruña y Pontevedra could also be attributed to the demographic structure of the population. In this sense, these provinces have a greater number of child patients [11], who generally have unhygienic habits, such as onychophagia, geophagy, or sucking on the thumb or other objects, which facilitate infection.

Although in our study we lack specific data on them, other factors that may also determine the variability in the consumption of antinematode drugs between the different Galician provinces are as follows: the number of doctors per inhabitant, the turnover rate of medical personnel, which could explain a wide individual variability in the prescription, and the profile of the patient to be treated, since the factors and habits of personal hygiene and basic domestic sanitation are significantly associated with the incidence of this type of parasitosis [14, 21].

This study presents a series of limitations that are common to DUS that apply the same methodology. Thus, dispensing without a medical prescription and prescription from the private sphere is unknown. However, it is estimated that this percentage should be low, at least in the case of mebendazole, due to the obligation to present a medical prescription for dispensing in pharmacy offices. Likewise, the prescription of this type of drugs through private entities, as well as the pharmaceutical indication of pyrantel, is marginal (María Antonia Cordeiro López's pharmacy and Carmen Goicoa Gago's pharmacy, personal communication), so it can be estimated that the exposed data provide an idea close to reality [21, 22].

## 5. Conclusion

This study has shown that mebendazole was the most consumed active principle in all the provinces and years studied. The main consequence of the overuse of this drug in comparison with pyrantel may be the increased risk of the development of resistance and of therapeutic failure, as well as the consequent limitation of pharmacological options in the future. The present work constitutes a first approach to the consumption of antinematode drugs in Galicia. Future studies are necessary to analyze consumption by age groups or sex, as well as the correct diagnosis of the disease and the appropriateness of the treatment, to better understand the determining factors of the consumption of this type of drugs.

## Figures and Tables

**Table 1:** Mean values and standard deviations (in italics) of the defined daily dose per 1000 inhabitants and day (DHD) by active principle and province (2016–2020).

	A Coruña	Lugo	Ourense	Pontevedra
Mebendazole	0.041 (*0.008*)	0.028 (*0.005*)	0.019 (*0.005*)	0.040 (*0.006*)
Pyrantel	0.002 (*0.0004*)	0.003 (*0.001*)	0.007 (*0.002*)	0.005 (*0.001*)

**Table 2:** Mean value of the defined daily dose per 1000 inhabitants and day (DHD) of mebendazole and pyrantel in each season (2016–2020).

	Spring	Summer	Fall	Winter
Mebendazole	0.031	0.030	0.036	0.033
Pyrantel	0.004	0.003	0.004	0.005

## Data Availability

The data used to support the findings of this study are available from the corresponding author upon request.
